# Positron emission mammography (PEM): reviewing standardized semiquantitative method

**DOI:** 10.1007/s12149-013-0748-y

**Published:** 2013-07-02

**Authors:** Yayoi Yamamoto, Youichiro Tasaki, Yukiko Kuwada, Yukihiko Ozawa, Atsushi Katayama, Yoshihide Kanemaki, Katsutoshi Enokido, Seigo Nakamura, Kouichi Kubouchi, Satoshi Morita, Mutsumi Noritake, Yasuo Nakajima, Tomio Inoue

**Affiliations:** 1 Yuai Clinic, 1-6-2 Kitashinyokohama, Kohoku-Ku, Yokohama, Kanagawa 223-0059 Japan; 2 Department of Radiology, Breast and Imaging Center, St. Marianna University School of Medicine, 6-7-2 Manpukuji, Asao-ku, Kawasaki, Kanagawa 215-8520 Japan; 3 Department of Breast Surgical Oncology, Showa University School of Medicine, 1-5-8 Hatanodai, Shinagawa-ku, Tokyo, 142-0064 Japan; 4 Yokohama Breast and GI Hospital, 1-5-18 Tunashimanishi, Kohoku-Ku, Yokohama, Kanagawa 223-0053 Japan; 5 Department of Biostatistics and Epidemiology, Yokohama City University Graduate School of Medicine, 3-9 Fukuura, Kanazawa-ku, Yokohama, Kanagawa 236-0004 Japan; 6 Department of Diagnostic Radiology, Yokohama City University Medical Center, 4-57 Urafune-cho, Minami-ku, Yokohama, Kanagawa 232-0024 Japan; 7 Department of Radiology, St. Marianna University School of Medicine, 2-16-1 Miyamae-Ku, Kawasaki, Kanagawa Japan; 8 Department of Radiology, Yokohama City University School of Medicine, 3-9 Fukuura, Kanazawa-ku, Yokohama, Kanagawa 236-0004 Japan

**Keywords:** Lesion-to-background (LTB), Maximum PEM uptake value (PUVmax), Positron emission mammography (PEM), Semiquantitative analysis

## Abstract

**Purpose:**

To validate semiquantitative analysis of positron emission mammography (PEM).

**Methods:**

Fifty women with histologically confirmed breast lesions were retrospectively enrolled. Semiquantitative uptake values (4 methods), the maximum PEM uptake value (PUVmax), and the lesion-to-background (LTB) value (3 methods) were measured. LTB is a ratio of the lesion’s PUVmax to the mean background; LTB1, LTB2, and LTB3 (which were calculated on different background) were used to designate the three values measured. Interobserver reliability between two readers for PUVmax and the LTBs was tested using the interobserver correlation coefficient (ICC). The likelihood ratio test was used to evaluate the relationship between ICCs. Receiver operating characteristic (ROC) curves were calculated for all methods. Diagnostic accuracy in differentiating benign tissue from malignant tissue was compared between PUVmax and LTB1.

**Results:**

The ICC rate was 0.971 [95 % confidence interval (CI) 0.943–0.986] for PUVmax, 0.873 (95 % CI 0.758–0.935) for LTB1, 0.965 (95 % CI 0.925–0.983) for LTB2, and 0.895 (95 % CI 0.799–0.946) for LTB3. However, there were some technical difficulties in the practical use of LTB2 and LTB3. The likelihood ratio test between PUVmax and LTB1 was statistically significant (*p* < 0.001). ROC curves of the 4 methods had similar characteristics. The median PUVmax was 1.39 for benign lesions and 3.70 for malignant lesions. LTB1 was 1.92 for benign lesions and 4.78 for malignant lesions. Significant differences (*p* < 0.001) in both PUVmax and LTB1 were observed between groups.

**Conclusion:**

Due to its simplicity and reproducibility, PUVmax is superior to LTB as an indicator for PEM in semiquantitative analysis.

## Introduction

^18^F-Fluorodexyglucose (FDG) positron emission tomography (PET) is a molecular imaging method that reflects glucose metabolism. FDG PET has a high sensitivity and specificity for detection of malignant lesions in general [1]. However, breast cancer detection now requires the ability to demonstrate non-palpable, small (<1.0 cm), invasive, and in situ malignancies [2]. Most whole body PET (WB PET) scanners provide spatial resolution of around 0.5–1.0 cm. This capability is insufficient for the requirements needed to image breast cancer; therefore, FDG PET is not used for primary breast cancer detection. Tumors that are less than 1.0 cm in size and of a low-grade are significantly associated with false-negative FDG PET results. To overcome such limitations, dedicated PET scanners for breast imaging have been developed. Dedicated PET scanners for breast imaging are classified into 2 groups. The first group comprises positron emission mammography (PEM) systems that use limited-angle tomography with 2 planar or curved detectors; the second scanner group acquires fully tomographic images of the breast. PEM Flex Solo II (Naviscan PET Systems) belongs to the former group. It consists of two oppositely placed planar detectors that gently compress the breast. The advantages of this system include its higher spatial resolution, shorter imaging time, and reduced attenuation compared to WB PET [3]. It provides a reconstructed spatial resolution of 2.4 mm. In early reports, it showed both high sensitivity (90 %) and specificity (86 %) for evaluation of known breast cancer or suspicious lesions [4]. The sensitivity and specificity rates were reported to be almost the same as for breast MRI [5, 6].

The assessment of a lesion seen on PEM is based on both visual interpretation and semiquantitative uptake. Narayanan et al. [5] reported that visual interpretation of PEM images is easy with minimal training, regardless of experience in breast imaging. They conducted a multicenter trial using a lexicon analogous to that of the standardized breast imaging reporting and data system (BI-RADS). Thirty-six observers individually reviewed PEM images. Mean sensitivity, specificity, and the area under the curve (AUC) of the PEM lesion were 96 %, 84 %, and 0.95, respectively. Interobserver agreement for PEM findings (including focus, mass, non-mass, or no uptake) was moderate, with a kappa value of 0.57. Final assessments (including benign, probably benign, or suspicious for malignancy) were substantial, with a kappa value of 0.63.

To the best of our knowledge, semiquantitative comparative analysis of PEM had not been previously performed. Either the lesion-to-background (LTB) value, which is recommended by PET machine manufacturers and has thus been used in numerous studies [4, 7, 8], or the PEM uptake value (PUV) was used for semiquantitative analysis. LTB is a ratio of the lesion’s maximum PEM uptake value (PUVmax) to the mean background value, but the method for measuring the background is unspecified and varies from report to report. Although no absolute FDG uptake threshold for malignancy exists, more intense uptake is thought to reflect the presence of malignancy [7].

The purpose of this study was to evaluate semiquantitative analysis for PEM as a potential alternative to SUVmax in whole body PET systems.

## Materials and methods

All subjects gave written informed consent prior to study inclusion. This retrospective study was approved by the institutional review board of our clinic.

### Patients

Our database was retrospectively reviewed for patients who underwent PEM between July 2007 and May 2012. Fifty patients were included in this study: 48 were suspected of having or diagnosed as having breast cancer, while 2 underwent PEM solely for screening. Overall, 61 lesions from 57 breasts were histologically proven benign or malignant breast lesions. The diagnosis of malignancy was made by an operative specimen or needle biopsy, and the diagnosis of benign was made by needle biopsy or fine needle aspiration cytology (FNA). Benign lesions that were diagnosed by FNA were followed up for more than a year with mammography or ultrasound. Findings were judged as benign when no change or decrease was seen on the imaging findings. Either repeat FNA or CNB was planned when findings on the images were suggestive of malignancy. Both patients who received a biopsy within 2 weeks before PEM and tumors that were not visible on mediolateral oblique (MLO) view mammograms were excluded in this study.

Among the 50 patients, 28 had malignant lesions only, one had double cancer, 11 had benign lesions only, and 10 had malignant and benign lesions. Among the 61 lesions from 57 breasts, 40 were diagnosed as malignant, and 21 were diagnosed as benign. The mean size of the malignant lesions was 24.0 mm (range 3–119 mm), and the mean size of the benign lesions was 15.1 mm (range 3–37 mm). All patients were female, with a mean age of 49.7 (range 29–73) years. The summaries of the patients are shown in Table 1. The malignant lesions comprised nine ductal carcinomas in situ (DCIS), 28 invasive ductal carcinomas, and three special type carcinomas. Fifty lesions underwent needle biopsy or FNA before PEM. Nineteen lesions underwent biopsy 2–4 weeks before PEM, and 31 lesions underwent biopsy more than 4 weeks before PEM. For 11 lesions, biopsy was performed after PEM.

**Table 1:** Patients’ characteristics

	Malignant lesions		Benign lesions	
	Mean ± SD	*n*	Mean ± SD	*n*
Body weight (kg)	53.35 ± 9.00	40	55.42 ± 9.81	21
Height (cm)	157.38 ± 4.86		158.90 ± 5.25	
Lesion size (mm)	24.03 ± 20.88		15.19 ± 10.39	
Injected dose of FDG (MBq)	175.47 ± 36.64		176.42 ± 28.81	

### PEM scanning

All patients had PEM imaging preformed in a commercially available PEM unit (PEM Flex Solo II, Naviscan PET system). This PEM unit has 2 opposing γ-ray paddles used to immobilize the breast. The detectors scan across the FOV in the direction of their 6-cm dimension to cover up to 24 cm, making the maximum FOV of the system 24 × 16.3 cm. The detectors are constructed from 2 × 2 × 13 mm lutetium yttrium orthosilicate scintillation crystals coupled to positron-sensitive photomultiplier tubes. The detectors are mounted on an articulating arm that rotates to allow imaging from different views. One of the detectors (the compression detector) is motor-controlled to set the distance between the 2 detectors (the compression thickness). Manual adjustment of the compression thickness is also possible. The image reconstruction uses five iterations of a 3-dimensional list mode maximum-likelihood expectation maximization (MLEM) algorithm. The PEM Flex system generates in-plane images with a pixel size of 1.2 mm, matrix size of 136 × 200, and FWHM of 2.4 mm.

The PEM Flex Solo II is a limited-angle focal-plane tomography system (tomosynthesis). Tomosynthesis has been primarily used in radiographic imaging. The PEM detector geometry results in a collection of coincidence lines of response with limited angular sampling that can be used to reconstruct high-resolution images parallel to in-plane images, but not perpendicular to the detector faces. The underlying reason for this is that the detectors do not encircle the object, nor do they rotate to acquire the 360° angular sampling required for fully 3-dimensional tomography. Tomosynthesis is associated with well-known problems of quantitative inaccuracy and strongly anisotropic spatial resolution. Because of the spatial anisotropy, 2 orthogonal imaging views, for example, craniocaudal (CC) and MLO, are required to achieve high-resolution imaging in all the 3 dimensions. In this study, all patients underwent MLO and CC views in the sitting position. However, semiquantitative values were calculated using only the MLO image.

The PEM Flex Solo II reports image values using a parameter referred to as the PEM uptake value (PUV). PUV is calculated by the following formula: tissue concentration (mCi/g) × weight (g)/injected FDG dose (mCi). The PUV differs from the standardized uptake value, which is a standard metric used in whole body PET, in that the activity concentrations measured in the PEM images are not corrected for attenuated or scattered photons. Because of this discrepancy, the manufacturer advocates evaluating image lesions using a ratio of lesion PUV divided by background PUV, referred to as LTB rather than using PUV alone as an image metric.

Patients were asked to fast for 4–6 h before the administration of a mean of 176.5 MBq (range 144.5–297.9 MBq) of FDG. The mean blood glucose level was 89 mg/dL (range 73–124 mg/dL). Prior to the PEM scan, a WB PET scan was performed after an average of 53 min (range 45–66 min), and PEM was performed 87 min (range 56–113 min) after FDG injection. Bilateral MLO and CC mammographic views were acquired with the breasts in mild compression.

The acquisition time was 8 min. PEM produces a 12-slice tomographic image display; the slice thickness, which is equal to the compressed breast thickness divided by 12, ranged from 1.5 to 6.3 mm (mean 3.9 mm) in this study.

### Data analysis

Two radiological technologists independently measured semiquantitative uptake without clinical information using a workstation (MIMviewer PEM 1.0, MIM Software Inc., Cleveland, OH, USA). The lesions of interest that were histologically proven were preliminarily set by a radiologist.

**Fig. 1:**
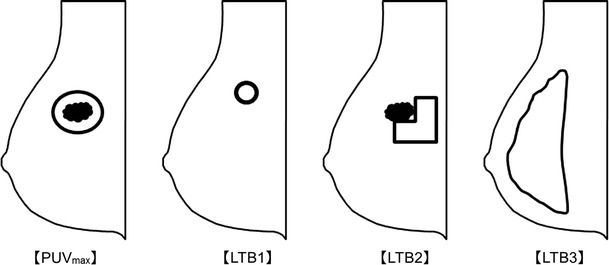
Methods for semiquantitative analysis. PUVmax: a region of interest (ROI) is drawn around the lesion in question and the maximum uptake is recorded as PUVmax. LTBs: the ratio of the lesion’s maximum PEM uptake value to the mean background is obtained. The mean background uptake for LTB1, LTB2, and LTB3 is determined in the following three ways: LTB1, a 2-cm circular ROI is drawn on the slice of nipple, and the ROI is drawn in a homogeneous area of normal breast tissue; LTB2, a 1-cm-wide L-shaped ROI is drawn adjacent to the lesion in question; and LTB3, a free-handed ROI is drawn on a slice of nipple, including all normal breast tissue. In cases of a *hotspot* on the slice of nipple, the area including the *hotspot* with a 1-cm margin is removed from the ROI

A region of interest (ROI) was drawn around the lesion in question, and the maximum uptake was recorded as PUVmax. LTBs, ratio of the lesion’s PUVmax to the mean background value, were then obtained. The mean background uptake for LTB1, LTB2, and LTB3 was determined in the following three ways: for LTB1, a 2-cm circular ROI was drawn on the slice of nipple, and the ROI was drawn in a homogeneous area of normal breast tissue; for LTB2, a 1-cm wide L-shaped ROI was drawn adjacent to the lesion in question; and for LTB3, a free-handed ROI, which included all normal breast tissue, was drawn on a slice of nipple (Fig. 1). In the case of a hotspot on a slice of nipple, the area containing the hotspot, along with a 1-cm margin, was removed from the ROI. The area within 1 cm of the chest wall was excluded from the background to reduce the edge effect artifact. PUV and LTB were measured by two analysts for the first 33 lesions, and the remaining 28 lesions were measured by one analyst.

### Analysis

Interobserver reliability between the two readers for PUVmax and LTBs was tested using the interobserver correlation coefficient (ICC). The likelihood ratio test was used to evaluate the relationship between ICCs.

Receiver operating characteristic (ROC) curves were calculated for all methods, and the AUC was determined using SPSS (Version 16.0, SPSS Japan Inc., Tokyo, Japan) and Rockit (Rockit 0.9B; C. Metz, University of Chicago, Chicago, IL, USA). The cut-off point between benign and malignant was calculated using Youden’s index [highest value for (sensitivity + specificity − 1)]. The performance on the PUVmax and LTB1 was compared for each histological diagnosis by the Mann–Whitney *U* test. In all analyses, *p* values less than 0.05 were considered significant.

## Results

### Interobserver variability

The ICC rate was 0.971 (95 % CI 0.943–0.986) for PUVmax, 0.873 (95 % CI 0.758–0.935) for LTB1, 0.965 (95 % CI 0.925–0.983) for LTB2, and 0.895 (95 % CI 0.799–0.946) for LTB3. Five cases were excluded from the LTB2 analysis because setting the L-shaped ROI was difficult. The likelihood ratio test between PUVmax and LTB1 was significant (*p* = 0.0009), but between PUVmax and LTB2 (*p* = 0.691) and between PUVmax and LTB3 (*p* = 0.04) it was not.

### ROC curve analysis

**Fig. 2:**
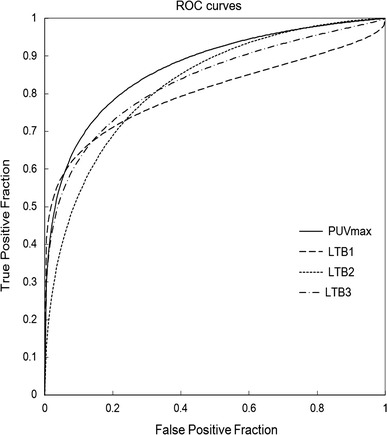
Receiver operating characteristic (ROC) curves of PUVmax, LTB1, LTB2, and LTB3. ROC curves of PUVmax, LTB1, LTB2, and LTB3 show almost the same characteristics. The area under the curve (AUC) for PUVmax is 0.87, which is higher than that for LTB1 (0.83), LTB2 (0.84), and LTB3 (0.84). Overall diagnostic performances for all 4 methods are moderate

ROC curves of PUVmax, LTB1, LTB2, and LTB3 are displayed in Fig. 2. All 4 curves showed nearly the same performance. The AUC for PUVmax was 0.86, which was higher than that for LTB1 (0.84), LTB2 (0.84), and LTB3 (0.85). Overall diagnostic performance for each of the 4 methods was moderate.

**Fig. 3:**
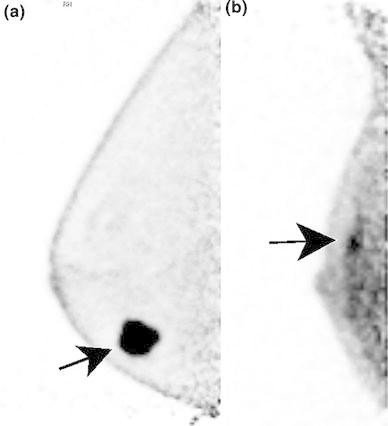
**a** A case with the same diagnosis with PUVmax and LTB1. The semiquantitative uptake is 10.52 for PUVmax and 34.89 for LTB1. Both PUVmax and LTB1 are higher than the cutoff calculated by the ROC curve in the present study, and the lesion is defined as malignant. *Mammography* shows an irregular mass in the fatty breast. Pathological result was invasive carcinoma. **b** A case with a different diagnosis with PUVmax and LTB1: a true positive for PUVmax and a false-negative for LTB1. Semiquantitative uptake is 3.10 for PUVmax and 1.68 for LTB1. PUVmax is higher than the cutoff calculated by the ROC curve in the present study, but LTB1 is lower. PUVmax suggests a malignant lesion, but LTB1 suggests a benign lesion. *Mammography* shows clustered amorphous calcifications in heterogeneously dense breast tissue. Mammography, ultrasound, and MRI reveal malignancy. Pathological result was DCIS

The cutoff point calculated by Youden’s index was 1.97 for PUVmax, with a sensitivity of 76 % and a specificity of 85 %. The cutoff point was 2.62 for LTB1 (sensitivity 76 %, specificity 85 %), 2.30 for LTB2 (sensitivity 82 %, specificity 70 %), and 1.97 for LTB3 (sensitivity 84 %, specificity 75 %). Between PUVmax and LTB1, one case defined as benign by PUVmax was defined as malignant by LTB1, and one case defined malignant by PUVmax was defined as benign by LTB1; the other 59 cases remained the same (Fig. 3).

### Lesion uptake

**Fig. 4:**
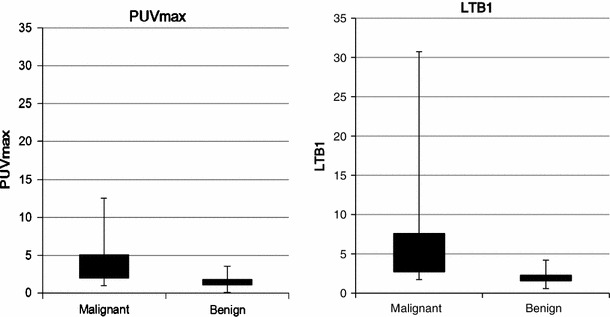
Uptake and findings of PUVmax and LTB1 (malignant vs. benign). A significant difference in the median PUVmax (*p* < 0.001) is seen between benign lesions (1.39, SD 0.70) and malignant lesions (3.70, SD 2.57). A significant difference (*p* < 0.001) is also seen in the median LTB1 between benign lesions (1.92, SD 0.91) and malignant lesions (4.78, SD 5.29)

A significant difference in the median PUVmax (*p* < 0.001) was seen between benign lesions (1.39, SD 0.70) and malignant lesions (3.70, SD 2.57). A significant difference (*p* < 0.001) was also seen in the median LTB1 between benign lesions (1.92, SD 0.91) and malignant lesions (4.78, SD 5.29) (Fig. 4).

**Fig. 5:**
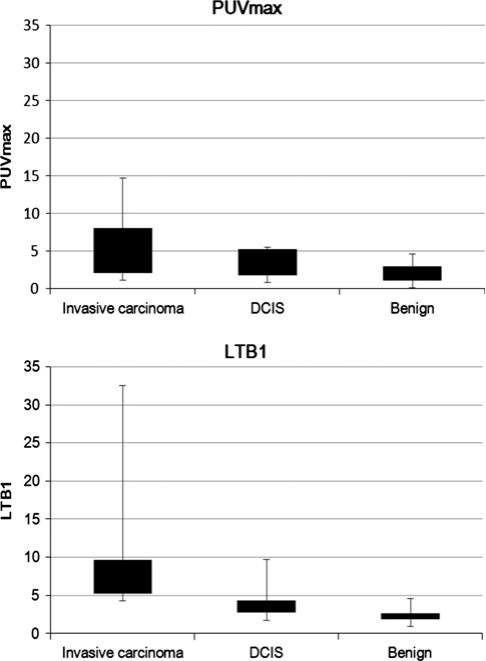
Uptake in findings of PUVmax and LTB1 (invasive carcinoma vs. DCIS vs. benign). Among the histopathologies of invasive carcinomas, DCIS, and benign lesions, substantial overlap is noted in both PUVmax and LTB1. The median PUVmax is 1.39 for benign lesions, 2.68 for DCIS, and 4.64 for invasive carcinoma. The median LTB1 is 1.92 for benign lesions, 2.75 for DCIS, and 5.25 for invasive carcinoma

Across benign, DCIS, and invasive carcinomas, substantial overlap was noted in both PUVmax and LTB1. The median PUVmax was 1.39 for benign lesions, 2.68 for DCIS, and 4.64 for invasive carcinoma. The median LTB1 was 1.92 for benign lesions, 2.75 for DCIS, and 5.25 for invasive carcinoma (Fig. 5).

## Discussion

In relation to PEM, PUV is not calculated with attenuation correction. Thus, the PET machine manufacturers recommend obtaining the LTB, and the usefulness of semiquantitative values is unclear. In the present study, LTBs were calculated three ways. However, there were some technical difficulties in the practical use of LTB2 and LTB3. In LTB2, it was difficult to set the L-shaped ROI in small breasts and in those with a large lesion. LTB3 involves a cumbersome procedure, since the ROI is set freehand. Therefore, the usefulness of PUVmax and LTB1 was compared in this study.

A significant difference was seen in the interobserver correlation between PUVmax and LTB1. PUVmax showed a higher correlation than LTB1. The cases that showed low correlations in LTB1s were breasts that were heterogeneously dense on mammography. In these cases, heterogeneously mixed fat and breast tissue may have led to heterogeneous FDG accumulation in the PEM images. The uptake of the background may easily change with different ROI areas.

In another study, Wang et al. [8] used PUVmax and LTB. The LTB for the two analysts showed a high correlation (0.98), which was better than the present study (0.87) for LTB1. The reason for this difference may be the breast size, racial differences, or patient age. This study consisted of Japanese women who tend to have smaller average breast sizes than Caucasian women [9]. Furthermore, the subjects were of a slightly younger mean age (49 years) than those in the study by Wang et al. (54 years). Small breasts might be affected by an artifact called edge artifact, which appears at the chest wall side of the image [2]. The edge artifact is an increased coefficient of variation (COV) within 1 cm from chest wall due to limited coincidence-count sampling at these positions. Thus, the PUVmean may easily change in these areas. Though these areas were excluded, some cases, especially patients with small breasts, might be affected by the edge artifact. Both race and age may affect breast density. del Carmen et al. [10] reported that the breast density was greater in Asian women than in both African–American and Caucasian women when not controlled for BMI and age. Young women tend to have dense breasts while older women tend to have fatty breasts. On PEM images, glandular breast background FDG uptake corresponds to breast density in mammography. The present study included more patients with dense and/or more heterogeneously dense breast tissue than the study by Wang et al., and this difference might have led to the different coefficients between the two studies.

Among PUVmax and all LTBs, the accuracy of the semiquantitative values was almost the same, since no differences were seen in the area under the ROC curve (AUC; 0.84–0.86). Although PUVmax may not accurately reflect a full quantitative recovery of counts to estimate FDG accumulation, this semiquantitative method had moderate accuracy, equal to that of LTBs. However, two types of cases, patients with extremely dense or fatty breast tissue, had different diagnoses. In such cases, the shape or distribution of hotspots and other modalities might help to clarify the diagnosis.

In other studies, Berg et al. [4] reported that when 2.0 was set as the cutoff for PUVmax, 53 % of malignant lesions was misdiagnosed as benign. That figure was worse than in the present study (with 1.7 cutoff for PUVmax, 23 % misdiagnosed as benign). Narayanan et al. [7] reported a significant difference between histology and a semiquantitative value. They reported a median value of PUV max as follows: benign 1.0, DCIS 1.1, and invasive cancer 1.4. For LTB1, they reported: benign 2.0, DCIS 2.5, and invasive cancer 2.3. The present study showed a much more distinct difference than Narayanan et al. for both PUVmax and LTB1. The wide distribution of tumor size in the present study was thought to be the reason for this difference. The mean size of the malignant lesion was larger in the present study than in the study by Narayanan et al. [7]. Numerous overlaps were observed between benign lesions, DCIS, and invasive cancer. In particular, most DCIS showed overlap between invasive cancer and benign lesions. The differential diagnosis between DCIS and benign lesions should be made carefully.

In WB PET, the highest or maximum standardized uptake value at one pixel (SUVmax) has been commonly used for analysis. In SUV, attenuation correction is needed because of marked differences in tissue density (e.g., lung vs. bone). However, in PEM, although extremely dense breast tissue may attenuate slightly more than fatty breast tissue, such differences are expected to be relatively minor with positron emitters in the breast [7]. Furthermore, PEM uses two opposing flat detectors, while WB PET uses full ring geometry. Due to the physics of the limited-angle tomography used in PEM, there is difficulty in accurately quantifying becquerels of FDG uptake per cubic centimeter of tissue [4]. Differences in reconstruction algorithms ensure that quantitation of PUV differs from that of SUV. In the present study, the relationship between SUVmax on WB PET and PUVmax on PEM was not examined because some lesions were too small to detect on WB PET. Wang et al. [8] reported that there is a high to moderate correlation between SUVmax and PUVmax or LTB.

Among the 4 analyses, PUVmax was a simple and reproducible indicator for PEM. When comparing the validation of LTB and that of PUVmax, PUVmax was superior to LTB as a semiquantitative analytic method for PEM. PUVmax and LTB1 showed the same diagnostic performance for distinguishing benign and malignant lesions.

This study has some limitations. In 50 lesions, PET was performed after biopsy, which might have affected the semiquantitative values. In order to minimize this effect, patients who had had a biopsy within 2 weeks of the PEM study were excluded. Another limitation is the standard reference. Two lesions diagnosed as benign by FNA were followed for more than a year with mammography or ultrasound, but another histological or cytological examination was not performed unless the lesions were suspected to be malignant. Therefore, some malignant lesions might have been judged as benign due to sampling error and their slow-growing nature.

In conclusion, PUVmax was suitable for standardized analysis. PUVmax is a simple technique with high interobserver correlation. In addition, semiquantitative values were shown to be useful for determining diagnostic characteristics in breast lesions.
